# Breather soliton dynamics in microresonators

**DOI:** 10.1038/ncomms14569

**Published:** 2017-02-24

**Authors:** Mengjie Yu, Jae K. Jang, Yoshitomo Okawachi, Austin G. Griffith, Kevin Luke, Steven A. Miller, Xingchen Ji, Michal Lipson, Alexander L. Gaeta

**Affiliations:** 1 Department of Applied Physics and Applied Mathematics, Columbia University, New York, New York 10027, USA; 2 School of Electrical and Computer Engineering, Cornell University, Ithaca, New York 14853, USA; 3 School of Applied and Engineering Physics, Cornell University, Ithaca, New York 14853, USA; 4 Department of Electrical Engineering, Columbia University, New York, New York 10027, USA

## Abstract

The generation of temporal cavity solitons in microresonators results in coherent low-noise optical frequency combs that are critical for applications in spectroscopy, astronomy, navigation or telecommunications. Breather solitons also form an important part of many different classes of nonlinear wave systems, manifesting themselves as a localized temporal structure that exhibits oscillatory behaviour. To date, the dynamics of breather solitons in microresonators remains largely unexplored, and its experimental characterization is challenging. Here we demonstrate the excitation of breather solitons in two different microresonator platforms based on silicon nitride and on silicon. We investigate the dependence of the breathing frequency on pump detuning and observe the transition from period-1 to period-2 oscillation. Our study constitutes a significant contribution to understanding the soliton dynamics within the larger context of nonlinear science.

## Abstract (web summary)

Breather solitons can be found in both physical and biological nonlinear systems. Here, Yu *et al*. demonstrate this type of soliton in silicon and silicon nitride microresonators, which advances the understanding of soliton-based comb-generation in microresonators.

Temporal cavity solitons (CSs) are self-localized pulses of light that can be excited in nonlinear optical resonators^1^,^2^,^3^,^4^,^5^,^6^,^7^,^8^ and have recently attracted significant research interest in the context of microresonator-based frequency comb generation^4^,^5^,^6^,^7^,^8^,^9^,^10^,^11^,^12^,^13^,^14^,^15^,^16^,^17^,^18^. In contrast, a breather soliton is a nonlinear wave in which energy is localized in space but oscillates in time, or vice versa^18^,^19^,^20^,^21^,^22^,^23^,^24^, and is found in various subfields of natural science, such as solid-state physics, fluid dynamics, plasma physics, chemistry, molecular biology and nonlinear optics^25^. Breather solitons have been recently reported in an optical fibre cavity^20^.

In this paper, we present a theoretical and experimental study of breather CS's^19^,^20^,^21^,^22^ excited in microresonators. This work provides experimental observation and characterization of such dynamic instabilities in optical microresonators^18^,^20^,^21^,^22^,^23^,^24^, which constitute a significant contribution toward understanding the universal dynamics of frequency combs based on driven passive resonators and is relevant to a large variety of physical systems for both fundamental and applied interests. We demonstrate the universal nature of such breather solitons in two different material platforms, silicon nitride (Si_3_N_4_)^9^,^10^,^12^,^13^ and silicon (Si)^16^,^17^. Our results establish a direct link between the breathing frequency and the pump-cavity detuning that is used to excite temporal CSs in microresonators.

## Results

### Numerical simulation of breather solitons

Temporal CSs were first studied experimentally in fibre cavities^3^,^20^,^26^,^27^ and subsequently reported in optical microresonators^4^,^5^,^6^. Mathematically, temporal CSs are a steady-state localized solution of the Lugiato–Lefever equation (LLE)^28^, which has been extensively used to model-driven nonlinear optical resonators^2^,^3^,^19^,^20^,^21^,^22^,^23^,^24^,^29^,^30^,^31^. The LLE allows for modelling of rich cavity dynamics, including a variety of instabilities and in particular, predicts the existence of persistent breathing CSs in optical resonators pumped by a continuous-wave (cw) laser^20^,^21^,^32^,^33^.

**Figure 1:**
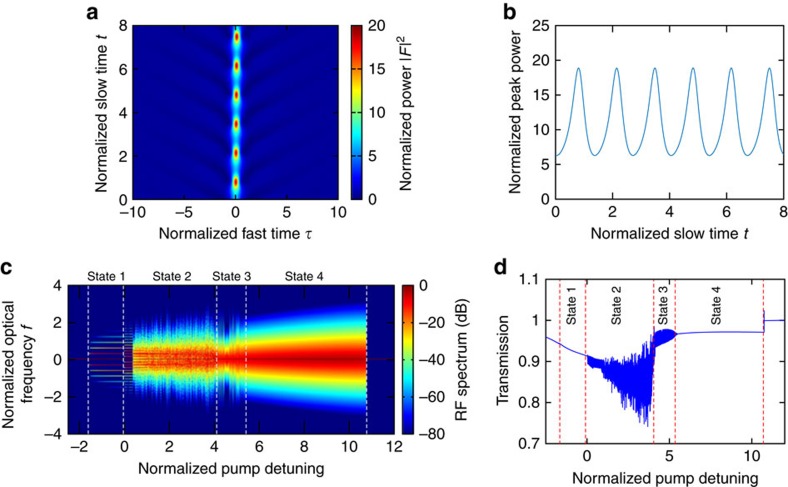
Numerical simulation of breather solitons. (**a**) The temporal evolution of a breathing temporal cavity soliton (CS). The period of the breathing cycle is 1.34 normalized slow time units. (**b**) The corresponding temporal evolution of the CS peak power. (**c**) Simulated density plot of instantaneous optical spectra as a function of the normalized absolute pump detuning Δ (from −2.9 to 12) and (**d**) the corresponding resonator transmission. The vertical dashed lines in **c**,**d** indicate the four different regions that correspond to (i) the primary comb state, (ii) unstable MI state, (iii) breather soliton state and (iv) stable soliton state. All the values are dimensionless (details in Methods).

In Fig. 1a,b, we simulate the temporal evolution of a breathing CS and its peak power evolution, respectively, in a microresonator using a normalized version of the LLE (for all details on numerical simulation, see Methods). Here we fix the dimensionless pump power *X*=8.7 and the cavity detuning Δ=4.5 and use the approximate analytic solution^2^ of a CS as the initial seed. It can be seen in Fig. 1a that as the CS evolves from bottom to top, it undergoes a periodic oscillation in time, which is accompanied by the associated breathing of the CS spectral envelope with the same period^22^. Furthermore, we present the evolution of the optical spectrum and the cavity transmission in Fig. 1c,d, respectively, as Δ is ramped up from −2.9 to 12, this time seeding the simulation with a noise corresponding to one photon per frequency mode. Such a detuning ramp is consistent with the typical experimental technique where the pump laser frequency is tuned across the resonance starting from the effectively blue-detuned regime (with respect to the thermally shifted hot resonance)^4^,^27^. We observe that the intracavity field initially develops into a low-noise primary comb state (State 1), destabilizes to an unstable modulation instability (MI) state (State 2), and eventually settles into the soliton state (State 4). This transition into the soliton state coincides with the pump frequency crossing the effective zero detuning and is accompanied by an abrupt change in the cavity transmission^4^ as demonstrated in Fig. 1d. More importantly, the breather soliton state (State 3) is identified by observing a periodic oscillation in time similar to Fig. 1a,b, and is located in the region of detunings between the unstable MI state and the stable CS regime, which is consistent with previous theoretical studies^20^,^21^,^22^,^23^,^24^.

### Experimental observation of breather solitons

**Figure 2:**
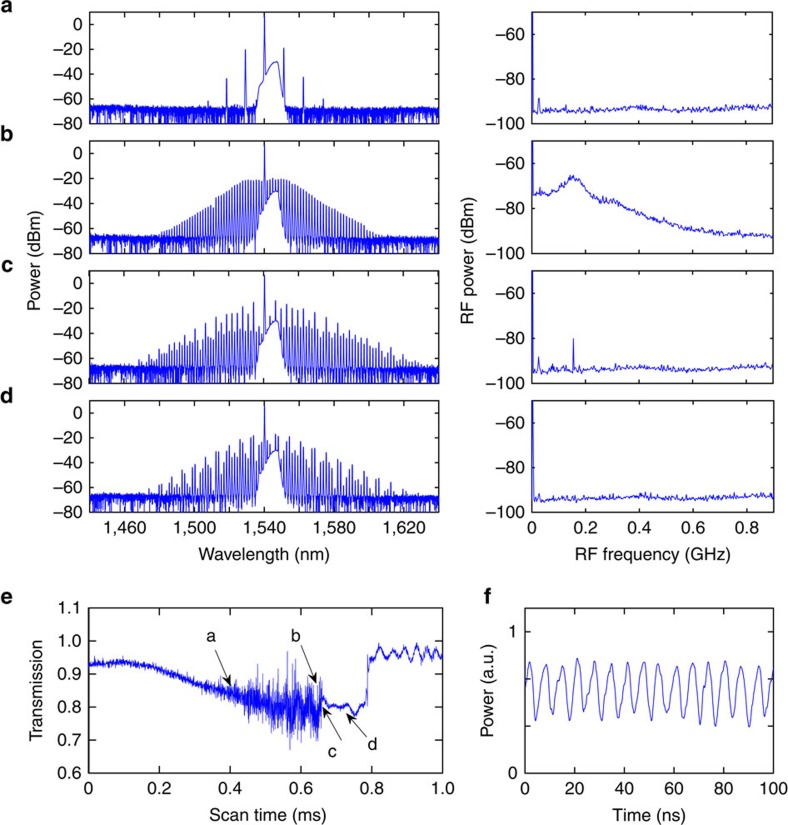
Observation of breather solitons in Si_3_N_4_ microresonators. (**a**–**d**) Optical and RF spectral evolutions where the state (**c**) shows breather solitons in Si_3_N_4_. (**e**) The pump power transmission as the laser frequency is scanned across the resonance. The scanning window corresponds to an absolute pump detuning range of 17 GHz. The arrows correspond to the four regions (**a**–**d**). The transmission step is indicative of soliton formation. (**f**) The recorded time trace of the comb output power at the breather soliton state (**c**).

In our experiment, we observe breather solitons in Si_3_N_4_ microresonators in the telecommunication wavelength range and in Si microresonators in the mid-infrared (mid-IR) range. In a Si_3_N_4_ microresonator, the spectral evolution towards soliton formation in both the optical and radio-frequency (RF) domains is plotted in Fig. 2a,d, as the pump laser is tuned into resonance. These four pairs of plots, from top to bottom, correspond to the generation of primary comb lines, unstable MI state, breather solitons and stable solitons, as demonstrated numerically in Fig. 1. Soliton formation is confirmed by low RF noise (Fig. 2d) and the abrupt cavity transmission step (Fig. 2c,d) in Fig. 2e. The time evolution of the comb output power for the breather soliton state is recorded in Fig. 2f, which shows the temporal oscillatory behaviour with a time period of 6.3 ns, which corresponds to four times the cavity photon lifetime and the RF beat note of 155 MHz in Fig. 2c. We attribute this sharp RF beat note to the occurrence of breather solitons, as will be further demonstrated in the next Section. Note that while such an RF peak has been observed previously in MgF_2_ resonators^4^ and Si_3_N_4_ microresonators^12^, it was not linked to the presence of breather solitons.

**Figure 3:**
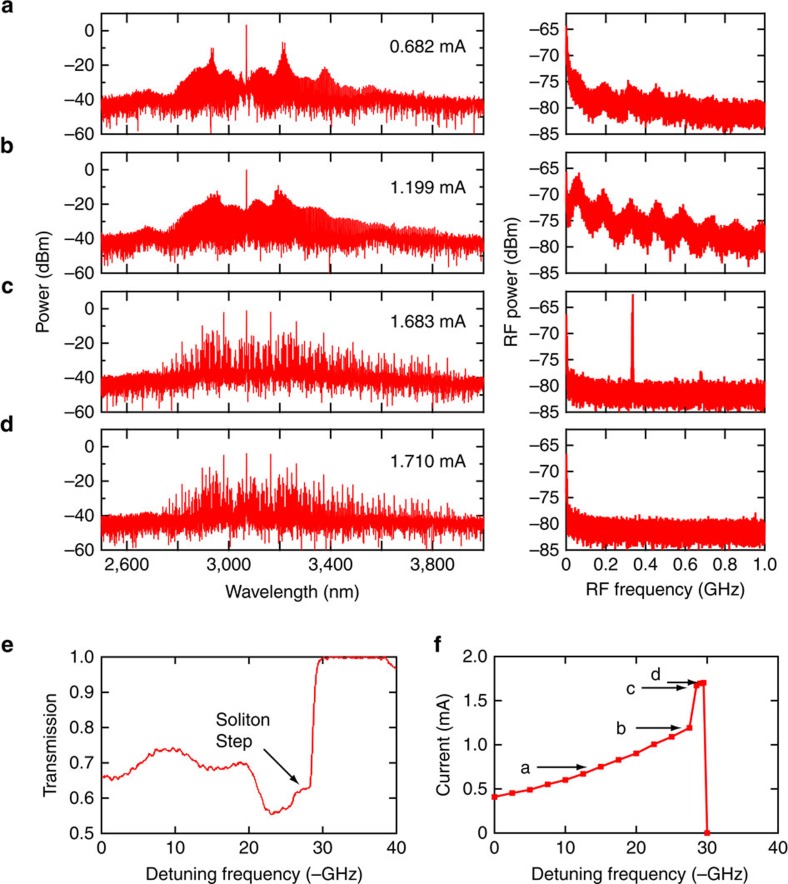
Observation of breather solitons in Si microresonators. (**a**–**d**) Optical and RF spectral evolution where the state (**c**) shows breather solitons in Si. Optical spectrum in **d** spans 0.8 of an octave (from 2.4 to 4.3 μm). No other significant features are observed in the RF spectra beyond 1 GHz up to 12 GHz. (**e**) The pump power transmission in Si as the laser frequency is scanned across the resonance. (**f**) Three-photon-absorption-induced current measured by scanning the pump detuning. Arrows correspond to four states (a–d), which are (a,b) unstable modulation instability states, (c) breather soliton and (d) stable soliton states.

In our Si microresonators, we monitor and analyse different states of the generated frequency combs by measuring the three-photon-absorption- (3PA-) induced photocurrent extracted from the integrated PIN junction, which is an effective way to probe the temporal behaviour inside a cavity^17^ (see Methods). Figure 3a,d shows the evolution of the generated optical and RF spectra as the pump laser is tuned into resonance in the same direction as in Si_3_N_4_ microresonators. Initially, from Fig. 3a,b), the d.c. component of the 3PA-induced current gradually increases due to intracavity power build-up. These spectra correspond to the unstable MI regime in which multiple independent mini-combs grow from the primary sidebands and spectrally overlap with each other leading to strong and broad RF beat notes^13^. Next, we observe a transition to a state (Fig. 3c) with a more structured optical spectrum and an abrupt increase in the measured d.c. current from 1.199 to 1.683 mA. The corresponding RF spectrum shows a sharp peak at 336 MHz with a low-noise background. Tuning the pump frequency further leads to a low-noise RF spectrum without any peaks (Fig. 3d). The variation in the d.c. current with pump detuning is shown in Fig. 3f. The sudden increase in the d.c. current from Fig. 3b,c indicates the formation of solitons with a high peak power (see Methods). We further confirm this by observing the concurrence between the abrupt current increase and a cavity transmission step^4^,^5^ in Fig. 3e. Thus, the state with the observed sharp RF peak (Fig. 3c) corresponds to a breather soliton state that exists beyond the unstable MI state but before the stable soliton state, which is consistent with our Si_3_N_4_ results, and agrees with our simulations and past theoretical studies^20^,^21^. Here the soliton breathes at a rate of 336 MHz, which corresponds to a period of 372 cavity roundtrips and 10 times the cavity photon lifetime. The narrow-linewidth RF beat note corresponding to the breather soliton state can be distinguished from the sharp RF beat notes observed at the onset of the unstable MI state^13^ since each one occurs at a different stage of comb evolution. The two states have drastically different temporal behaviour (for example, temporally localized pulse formation in the breather soliton state but not in the unstable MI state), which is easily detected by our technique here by measuring the 3PA-induced photocurrent.

The excitation of breather solitons in both platforms requires a certain range of pump power. For example, we observe an evolution towards a stable soliton state without a breather soliton state at a relatively low pump power and more complex dynamics at a higher pump power. In addition, it can be achieved by tuning the pump frequency into resonance without changing the pump power^33^.

### Characterization of breather soliton dynamics

**Figure 4:**
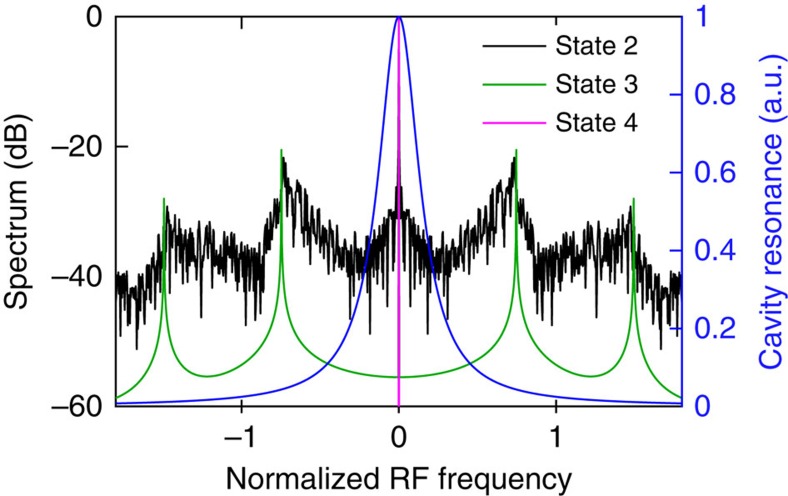
Numerical simulations of RF spectra at selected detunings. Specific detuning values are Δ=3.9 (State 2), Δ=4.5 (State 3) and Δ=8 (State 4). For comparison, the plot also shows the loaded Lorentzian resonance curve (blue curve; right axis). Note that the RF frequency corresponds to the normalized slow time that is dimensionless. For details on simulations, see Methods.

Another important aspect of breather soliton dynamics is that during the transition into the breather soliton state, the broad RF beat note corresponding to the unstable MI state becomes significantly narrower while maintaining the same centre frequency, as shown in Fig. 2b,c. Such evolution is also observed in some Si microresonators for which the higher order harmonics of the 3PA-induced current noise do not obscure this feature. This sudden narrowing of the RF beat note is also well predicted by our numerical simulations of the RF spectra of the comb transmission at different comb states corresponding to Fig. 1c,d. Our modelling indicates that the breathing CS manifests itself through low-frequency (relative to the comb spacing) modulation sidebands in the RF domain^18^, as shown in Fig. 4 (green curve) at the pump detuning corresponding to State 3 in Fig. 1c. In addition, the simulated RF spectra at two other pump detunings (States 2 and 4 in Fig. 1c) are plotted in Fig. 4 for comparison. It can be seen that at the detuning Δ=3.9 (State 2; black curve), the system is initially in an unstable MI state characterized by a broad primary noise peak. In the time and spectral domains, the intracavity field fluctuates significantly from one roundtrip to another, analogous to spatiotemporal chaos originally discussed in the context of plasma physics^32^. In contrast, as the detuning is increased past a threshold value, the RF noise peak abruptly sharpens while maintaining its central frequency (State 3: Δ=4.5; green curve). Note that the simulated breathing period is 2.7 times the cavity photon lifetime. For comparison, on the same plot, we also show the loaded Lorentzian resonance curve of the resonator under consideration on the same axes (right axis; blue curve). The sharpening of the RF beat note can be mathematically explained as follows. Just before the system crosses the boundary from the unstable MI regime to the breather state, the eigenmode associated with the breather state corresponds to the complex eigenvalue with the largest positive real part (that is, the most unstable). As the system enters the breather state, this eigenmode dominates the cavity dynamics while other unstable modes become stable and dampen out over time. As a result, the beat note frequencies of the two distinct states are expected to coincide. The sideband eventually drops to a negligible level with an increased detuning (State 4: Δ=8; magenta curve), which signifies the stabilization of the temporal CS, where the pulse reaches a time-stationary state.

**Figure 5:**
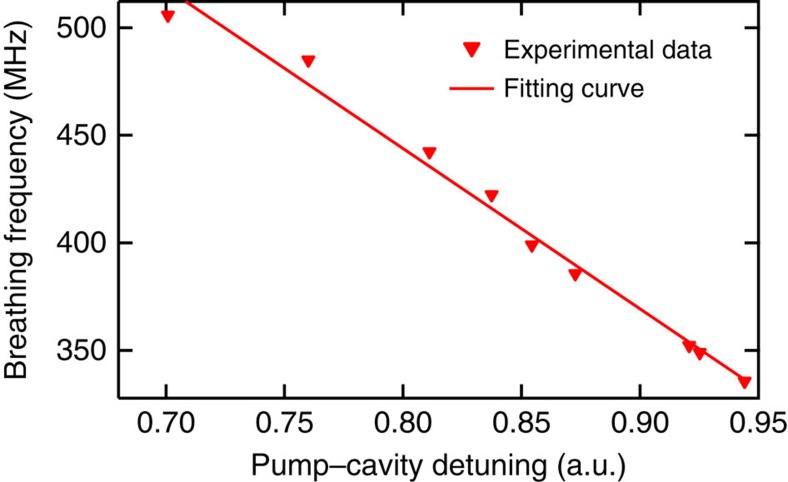
Breathing frequency dependence on the effective pump-cavity detuning. The effective pump-cavity detuning in the Si microresonator is extracted from the three-photon-absorption-induced photocurrent based on the relationship with the soliton peak power and thus in arbitrary units (a.u.). The same trend is also observed in a Si_3_N_4_ microresonator.

Next, we investigate the factors that influence the breathing frequency. We find that the breathing frequency can be continuously tuned by changing the effective pump-cavity laser detuning. By tuning the pump to shorter wavelength while the soliton state is maintained, the effective pump detuning decreases, and we observe a linear increase in oscillation frequency as well as a decrease in modulation depth in both platforms. In other words, the breathing frequency decreases linearly with an increasing effective pump-cavity detuning as shown in Fig. 5 for Si microresonators. For comparison, we run a series of numerical simulations based on the normalized LLE (Methods) and present the detuning dependence of the breathing frequency for three different pump power levels in Supplementary Fig. 1. In the lower detuning range near the transition point between the unstable MI and the breather states, the breathing frequency either exhibits minimal change (lower pump power) or decreases linearly (higher pump powers) with an increasing detuning. The latter trend is consistent with our experiments in which the pump frequency is tuned past the transition point with increasing Δ. This may hint that we are operating at relatively high pump powers. In addition, our numerical simulations reveal that in the higher detuning range, a linear increase in the breathing frequency is expected with increasing detuning. However, this trend is not currently observed in our experiments and is under further investigation.

**Figure 6:**
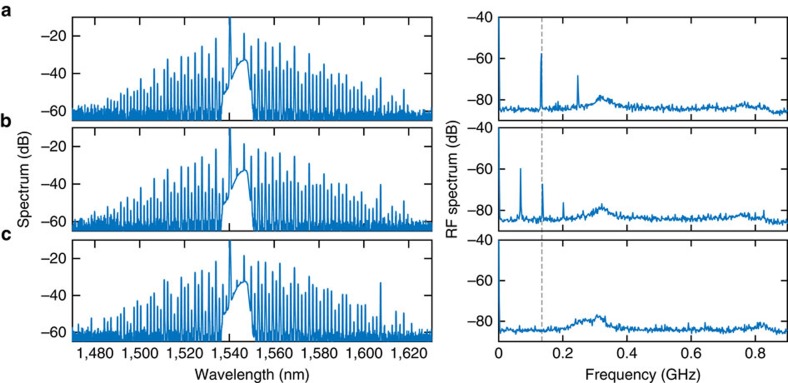
Temporal cavity soliton of higher oscillatory periodicity in a Si_3_N_4_ microresonator. At a higher pump power, as the pump detuning increases, the period-1 oscillation (**a**) transitions to a period-2 oscillation (**b**), and the cavity soliton finally stabilizes (**c**). The dashed line indicates that the first RF beat note in state (**a**) aligns with the second RF beat note in state (**b**) at the transition point.

Microresonators allow access to larger detuning values in the cw regime (cf. quasi-cw synchronous pumping^33^) due to their high finesse. At higher values of the detuning and pump power, more complicated dynamical regimes can be found in the LLE, such as period-2 oscillations, period-*N* oscillations and temporal chaos^20^,^33^. In the Si_3_N_4_ microresonator, using a higher pump power than that in Fig. 2, we observe a transition from the period-1 oscillation to the period-2 oscillation (Fig. 6a,b), and the field finally stabilizes into stable CSs (Fig. 6c) as the pump detuning is increased. Unlike the theoretical prediction of ref. 20, we do not observe the system returning to the period-1 breather state beyond the period-2 regime. A possible explanation is that the thermal effect acts as an additional source of instability that hinders our observation of the expected behaviour, especially across the boundaries of different regimes. Nevertheless, this is the first time an oscillatory temporal CS with higher periodicity has been observed experimentally.

## Discussion

In conclusion, we show the universality of the dynamics of the breather CS formation in microresonators, attributing the formation of narrow low-frequency RF modulation sidebands in the modelocked CS regime to time-oscillating behaviour of CSs. The universal nature of breather soliton formation is indicated by our observation in two distinct platforms that have different material characteristics, device geometries and operating conditions. Our results present experimental confirmation of the pioneering theoretical studies of a breather soliton solution in a cw-pumped high-finesse microresonator and provide experimental techniques for their observation, which agrees well with our numerical results. Our work also indicates that the breathing frequency is dependent on the pump-cavity conditions, and reveals that microresonators can be an ideal test bed for fundamental theories of nonlinear wave dynamics that are relevant to a large variety of physical systems.

## Methods

### Numerical simulations

A variety of dynamical regimes in driven passive resonators can be modelled based on a modified LLE:


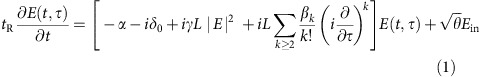


Here *E*(*t*, *τ*) is the slowly varying intracavity field envelope, *t* is the slow evolution time on the order of the cavity roundtrip time *t*_R_, while *τ* is a fast time related to the group velocity of light inside the resonator. *α* is a fraction corresponding to half the total percentage power loss per roundtrip and *L* is the cavity length. *γ* is the nonlinear parameter, and *β*_*k*_ is the *k*th order dispersion coefficient. Finally, *θ* is the input coupling coefficient, *E*_in_ is the pump-field strength, and *δ*_0_ is the phase detuning of the pump with respect to the nearest cavity resonance.

To ensure broader applicability of our study, we employ the following dimensionless form of the LLE^3^


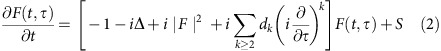


Here we have used the normalization convention 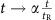, 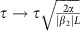, 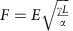, 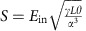, 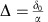, with the additional expression for the normalized dispersion coefficients 
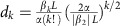
, such that the second-order dispersion coefficient *d*_2_ represents the sign of the group-velocity dispersion (*d*_2_=−1 in our case). The optical frequency *f* and RF frequency used in Figs 1 and 4 correspond to the dimensionless fast time *τ* and slow time *t*, respectively, and hence are also dimensionless. Note that *S* (equivalently, *X*=|*S*|^2^) and Δ are the normalized pump-field strength (pump power) and detuning, respectively, and are the only control parameters of the current system under consideration, thus simplifying our investigation. For specificity, however, we use parameters similar to our 200 GHz free-spectral-range (FSR) Si_3_N_4_ resonator with 950 × 1,500 nm cross-section, that is, *t*_R_=FSR^−1^=5 ps, *α*=0.0018, *θ*=0.0005, *γ*=0.9 W^−1^m^−1^, *L*=0.63 mm, *β*_2_=−202 ps^2^ km^−1^, *β*_3_=0.034 ps^3^ km^−1^, *β*_4_=7.7 × 10^−4^ ps^4^ km^−1^ and |*E*_in_|^2^=170 mW. Depending on the simulation, we vary *δ*_0_ from −0.00522 to 0.0216, rad. These parameters correspond to |*S*|^2^=*X*=8.7 and Δ=−2.9 to 12, *d*_2_=−1, *d*_3_=0.003 and *d*_4_=9 × 10^−6^. Although we include higher-order (third and fourth) dispersion coefficients for completeness, their effects are negligible and do not invalidate the applicability of the past theoretical studies to our case.

The LLE is integrated using the split-step Fourier method. In Fig. 1c,d, we seed the initial parametric process with the noise consisting of one photon per mode with random phase. The normalized pump power *X*=8.7 is used and the detuning Δ is varied from −2.9 and 12 at a rate of 0.001 per one normalized slow time unit (one normalized time unit corresponds to two cavity photon lifetimes). The RF spectra in Fig. 3 are obtained by using the intracavity fields saved during the detuning scan simulation as the initial conditions, and regularly sampling the roundtrip-averaged out-coupled power 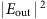 (measured in real unit W), where 
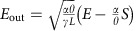
. For each detuning, before sampling, we numerically propagate the intracavity field for a sufficiently long time (150 slow time units, or ∼300 cavity photon lifetimes) to allow the system to reach steady state (quasi steady state for breather soliton and unstable MI states). For Fig. 1a,b, we use the fixed pump power and detuning (*X*=8.7, Δ=4.5), and an approximate CS solution 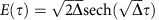 as the initial condition. It should be noted that during its evolution, the breather pulse and its associated temporal features (for example, pulsating ripples) remain sufficiently localized and does not cause complications arising from boundary effects.

Our numerical simulation exhibits a qualitative agreement with experimental results. Due to the thermo-optic effect in the microresonator geometry, the pump power (*X*) and detuning (Δ) are not completely decoupled. A direct quantitative comparison between numerical and experimental results should be possible if the thermal effect is carefully measured and taken into account in the model.

### Devices and experimental set-ups

Details for the device parameters are listed in Supplementary Table 1. For comb generation in one of the Si_3_N_4_ microresonators, which has a cross-section of 950 nm × 1,500 nm and an FSR of 200 GHz, we detune the pump laser at 1,540 nm into a cavity resonance with a bus waveguide power of 56 mW. We estimate a *Q* factor >2 million. To measure the RF spectrum, we filter out the pump wavelength using a wavelength-division multiplexing coupler and send the output to a photodiode. Similar breather soliton dynamics is also demonstrated in another Si_3_N_4_-microresonator that has a cross-section of 950 nm × 1,400 nm and an FSR of 500 GHz, and is pumped at 1,560 nm. The measured *Q* factor is 1.9 million.

Next, we utilize a high-*Q* (∼200,000) etchless Si microresonator with a device structure as described in ref. 16 with an FSR of 127 GHz. The resonator is pumped using a cw optical parametric oscillator (<100 kHz linewidth). The comb generation dynamics are characterized as the pump centred at 3.07 μm is tuned into a cavity resonance with an off-resonance bus waveguide power of 80 mW. To reduce the optical losses due to free carriers (FCs) generated from 3PA, we use an integrated PIN junction^16^ and apply a reverse bias voltage of −12 V. Simultaneously, the d.c. and RF components of the current extracted from the PIN are monitored using a Keithley SourceMeter and a RF spectrum analyzer, respectively. The measured RF current reflects noise properties of intracavity comb generation with a bandwidth of 12 GHz, which in this case is limited by the RF amplifier we use before the RF spectrum analyzer. More importantly, due to the intrinsic 3PA process, the FC is generated at a rate proportional to the cube of the optical intensity, yet with a decay rate determined by the FC lifetime of a specific PIN structure, expressed in the equation below^34^ (in real units):


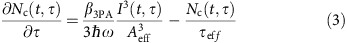


Here 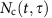, 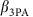, 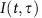, 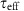 and 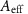 are the FC density, 3PA coefficient, optical intensity, FC lifetime and the effective mode area, respectively while *ω*, *t* and *τ* are the pump frequency, and fast and slow time scales, respectively. Typically, in an unstable MI state, the intracavity optical power is relatively low and features quasi-cw behaviour due to a low spectral coherence while a soliton state is associated with short pulses with high peak power on top of a low cw background within one roundtrip. Therefore, an increased 3PA-induced current is always expected at the transition to the soliton state. In addition, any fluctuation of intracavity power will result in the fluctuation of current at the fundamental frequency, along with its second and third harmonics. The fundamental frequency modulation depth will be enhanced by the square of the d.c. component of the current. In this sense, the 3PA-induced current is a powerful tool to probe the temporal behaviour of the generated frequency combs, especially in the soliton modelocked regime that features pulse formation with much higher peak power. The detection of the breather soliton state requires fast and sensitive measurement techniques in the mid-infrared, especially when the oscillating frequency is far off resonance or the modulation depth is not significant. Our technique based on the 3PA-induced current is experimentally proven to be an efficient way to observe these dynamics.

Note added: Concurrent with submission of this work, a related experiment was reported by Bao *et al*.^22^

### Data availability

The data that support the plots within this paper and other findings of this study are available from the corresponding author upon reasonable request.

## Additional information

**How to cite this article:** Yu, M. *et al*. Breather soliton dynamics in microresonators. *Nat. Commun.* **8,** 14569 doi: 10.1038/ncomms14569 (2017).

**Publisher's note:** Springer Nature remains neutral with regard to jurisdictional claims in published maps and institutional affiliations.

## Supplementary Material

Supplementary Information Supplementary Figure, Supplementary Table, Supplementary Note and Supplementary References
