# Supplementary material for: Breather soliton dynamics in microresonators
Source: Nat Commun. 2017 Feb 24;8:14569. doi: 10.1038/ncomms14569 (PMC5333125; doi:10.1038/ncomms14569)
Supplement: Supplementary Information — Supplementary Figure, Supplementary Table, Supplementary Note and Supplementary References [file ncomms14569-s1.pdf]

Supplementary Note 1

In this Section, we numerically show the dependence of the breather frequency on the dimensionless cavity detuning Δ by employing the mean-field Lugiato-Lefever equation (LLE) presented in the Methods of the main text. We consider 3 different pump power levels[1]: just above the breather threshold, slightly below the period-2 oscillation threshold, and within the period-2 oscillation regime. For each pump power, we start from the lower detuning boundary of the breather regime, seed the simulation with an approximate analytic solution[2] of the stable temporal cavity soliton (CS) and numerically propagate the intracavity field over 10 normalized time units (20 cavity photon lifetimes). Then we regularly sample the roundtrip-averaged intracavity power over the subsequent 190 time units (380 cavity photon lifetimes) in order to obtain the RF spectrum. The breather frequency can be extracted from the resulting RF spectrum. We repeat this process for each detuning as the detuning is slowly ramped up with a step size of 0.2.

The results are summarized in Supplementary Figure 1. Overall, as consistent with the past theoretical studies[1], the breather regime shifts to higher detunings with an increasing pump power. Several other trends can also be seen. As the detuning increases, the breathing frequency initially does not change significantly (low power; green triangles), or even decrease linearly (higher powers; red squares and blue circles). Toward the higher detuning, the trend reverses and can be approximated by a straight line with a positive slope for each power. Our experiments are conducted by tuning the pump frequency in the increasing direction of Δ across the transition point from the unstable modulation instability to the breather states, and agree qualitatively with the trend observed at the lower detuning range. However, the positive linear trend toward the higher detuning range has not been observed in our experiment despite the

fact that it occurs over a larger detuning range. This behavior is currently under further investigation.

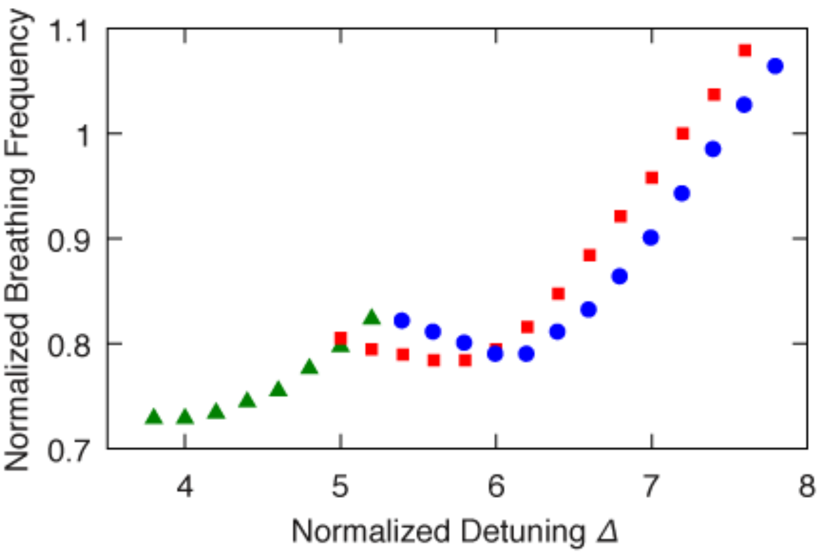

Supplementary Figure 1 | Simulation of the breathing frequency as a function of the normalized detuning (Δ) for 3 different pump power levels: just above the breather threshold (green triangles), slightly below the period-2 oscillation threshold (red squares), and within the period-2 oscillation regime (blue circles). Breathing frequency is normalized with respect to 0.5 * photon lifetime^{-1}. Detuning is normalized with respect to the cavity roundtrip loss.

Supplementary Table 1

Device parameters for the microresonators

Platform	*Q*-factor	Finesse	Linewidth	Free-spectral range	Photon lifetime
Si	~ 200,000	255	~ 500 MHz	127 GHz	~ 0.3 ns
Si_3N_4	~ 2,000,000	~2,000	~ 100 MHz	200 GHz	~ 1.6 ns

Supplementary References

1. Leo, F., Gelens, L., Emplit, Ph., Haelterman, M. & Coen, S. Dynamics of one-dimensional Kerr cavity solitons, *Opt. Express* **21**, 9180-9191 (2013).

2. Wabnitz, S. Suppression of interactions in a phase-locked soliton optical memory, *Opt. Lett.* **18**, 601-603 (1993).
